# Research on Diffusible Signal Factor-Mediated Quorum Sensing in *Xanthomonas*: A Mini-Review

**DOI:** 10.3390/molecules28020876

**Published:** 2023-01-15

**Authors:** Yu-Mei Feng, Zhou-Qing Long, Hong-Mei Xiang, Jun-Ning Ran, Xiang Zhou, Song Yang

**Affiliations:** 1 National Key Laboratory of Green Pesticide, Key Laboratory of Green Pesticide and Agricultural Bioengineering, Ministry of Education, Center for R&D of Fine Chemicals of Guizhou University, Guiyang 550025, China; 2 School of Chemistry and Chemical Engineering, Qiannan Normal University for Nationalities, Duyun 558000, China

**Keywords:** quorum sensing, enzyme inhibitor, resistance, DSF, RpfF

## Abstract

*Xanthomonas* spp. are important plant pathogens that seriously endanger crop yields and food security. RpfF is a key enzyme that is involved in the synthesis of diffusible signal factor (DSF) signals and predominates in the signaling pathway regulating quorum sensing (QS) in *Xanthomonas*. Currently, novel RpfF enzyme-based quorum sensing agents have been proposed as a promising strategy for the development of new pesticides. However, few reports are available that comprehensively summarize the progress in this field. Therefore, we provide a comprehensive review of the recent advances in DSF-mediated QS and recently reported inhibitors that are proposed as bactericide candidates to target the RpfF enzyme and control plant bacterial diseases.

## 1. Introduction

In the process of disease occurrence, enzymes, which are a class of catalytically active proteins, catalyze the production of regulators in pathological pathways. In particular, the targeting of enzymes is key to the development of novel agricultural agents [1]. The enzyme inhibitors applied in agriculture are mostly pesticides and herbicides (e.g., organophosphate insecticides that target acetylcholinesterase and glyphosate that targets 5-enolpyruvylshikimate 3-phosphate synthase) [2,3]. Meanwhile, research on fungicides has focused on the main enzyme targets involved in energy metabolism and biosynthesis, such as succinate dehydrogenase (SDH) and pyruvate kinase (PK) [4,5,6]. Conversely, agricultural bactericides are extremely limited and less well studied. The design of green agrochemicals using target enzymes has been an important strategy. Therefore, studying new target enzymes would be conducive to the development of new bactericides with high efficiency, low toxicity, and good specificity [7,8].

*Xanthomonas* spp., which belong to Gram-negative bacteria, are a class of plant pathogens that can infect at least 124 monocotyledonous and 268 dicotyledonous plant species, including a variety of important agricultural crops, causing huge losses in agricultural production [9]. In particular, *Xanthomonas oryzae* and *Xanthomonas campestris* are among the top ten phytopathogens of interest in plant molecular pathology [10]. *Xanthomonas campestris* pv. *campestris* (*Xcc*) infects cruciferous plants to cause black rot disease, which is periodically diffused as a worldwide plant disease, and *Xanthomonas oryzae* pv. *oryzae* (*Xoo*) infects rice and causes bacterial leaf disease, leading to rice yield losses ranging from 20% to 80% [11,12]. In addition, *Xanthomonas* spp. produce virulence factors that strengthen the competitive advantages and enhance the environmental adaptability for host plant infection and bacterial multiplication [13]. The key virulence factors include extracellular polysaccharides (EPSs) and extracellular enzymes, and their biosynthesis involves multiple coordinated regulatory systems [14].

**Figure 1:**
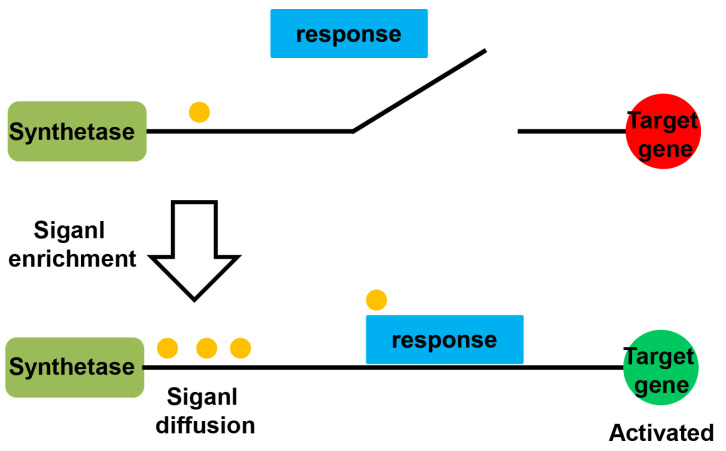
The quorum sensing pathway.

Since the original discovery of the bioluminescent properties of the bacterium *Vibrio fischeri* in 1968 and the concept proposed in 1994 by Fuqua et al., the quorum sensing (QS) system is increasingly being investigated [15,16]. QS is a communication process between bacteria that regulates virulent gene expression in response to recognizing population density, typically in association with the production and perception of signaling molecules called auto-inducers (AIs) [17]. The signal integration pathway mainly contains the following important components: a signal synthase, a signal receptor, and a signal transporter [18]. As displayed in Figure 1, at low cell densities or low concentrations of signaling molecules, QS is inactivated. When an accumulation of signal molecules gradually reaches a threshold due to the increase in a bacterial population, the QS pathway is triggered, and the activation of intracellular signaling pathways leads to a coordinated increase in gene expression to produce various virulence factors, including biofilms, extracellular enzymes, phytotoxins, and motility factors, thus providing favorable conditions for the bacteria to infect the host [19].

The most well-known QS systems are mediated by *N*-acyl homoserine lactones (AHLs), which are the first signaling molecules to be identified in Gram-negative bacteria [20]. Somewhat differently, the QS system for *Xanthomonas* is regulated by diffusible signal factors (DSFs) rather than AHLs [21]. The QS in *Xanthomonas* is also associated with rpf (regulation of pathogenicity factor) genes, among which the main genes *rpfF*, *rpfB*, *rpfC,* and *rpfG* encode proteins that are involved in DSF synthesis, turnover, sensing, and transduction, respectively [22]. Ordinarily, the inhibition of QS occurs via limiting signal accumulation. It is a widely studied approach that involves the degradation and deactivation of signaling molecules, especially in the AHL-mediated mode, such as quorum quenching enzymes [23]. Notably, an alternative approach is the inhibition of synthetase, which is studied less than the former and scarcely reported in DSF-mediated QS. However, some studies indicate that this approach is feasible, effective, and has the potential to inhibit plant pathogen infection processes [24].

The QS pathway is currently being considered as a target for drug design, and there are multiple regulatory proteins that may also participate as possible targets, such as signaling molecular synthases. This review aims to describe the QS pathway in *Xanthomonas* spp., illustrate the key role of the signaling molecule DSF and that of the biosynthase RpfF, and provide a summary of advancements in the research on RpfF enzymes as potential targets to control plant diseases.

## 2. Rpf/DSF Quorum Sensing

### 2.1. The rpf Gene Cluster

The regulation of the *rpf* gene cluster in relation to virulence factors has been widely studied in *Xanthomonas* spp. Generally, the *rpf* cluster of genes consists of at least nine genes (*rpfA*-*rpfI*) [25]. It has been reported that *rpfABFCHG* is in the left-hand section and rpfDEI is in the right-hand section [26]. In *Xcc*, *rpfA*, which is adjacent to *rpfB* and *rpfF*, is involved in the regulation of pathogenicity factor production and encodes an aconitase that is implicated in iron homeostasis [27]. Instead, *rpfB* and *rpfF* are involved in the synthesis of DSF in *Xanthomonas*. In *X. oryzae* pv. *oryzae* PXO99A, mutants with an *rpfB* deletion can also boost DSF production, and mutant *rpfB* overexpression can induce the opposite results [28]. In addition to encoding an enoyl-CoA hydratase, which is involved in DSF synthesis, the *rpfF* gene plays a prominent role in the maintenance of membrane integrity in *Xoo* by regulating the fatty acid synthesis pathway [29]. The mutant of *rpfF* exerts a negative influence on the *rpfB* gene and fatty acyl-CoA ligase (FCL) activity [30]. In *Xoo*, to define the function of the main *rpf* genes, *rpf* mutants were inoculated on rice. With regard to the lesion length, *rpfB*, *C*, *F*, and *G* had a more significant influence on the pathogenicity than *rpfA*, *D*, and *I* [31]. In *Xcc*, *rpfC* and *rpfG*, respectively, encode two proteins that together compose a two-component system, and *rpfH* is described as part of the *rpfGHC* operon, which encodes RpfC/RpfG, a two-component system [25,32]. The mutant *rpfC* led to the overproduction of DSF and lower levels of EPS and extracellular enzymes. Moreover, the *rpfC* mutant decreased the expression of *hrpX* and expressed a weakened phenotype of the hypersensitive response (HR), although the mutant *rpfG* did not affect *hrpX*. In other words, *hrpX* is important for the Type III secretory system (T3SS) and is positively regulated by *rpfC*, independently of *rpfG* [33]. Further studies are needed to shed light on the regulatory link between *rpf*/DSF and *hrp*/T3SS. Unlike the left-hand section, the right-hand section has fewer studies due to the minor regulatory roles. It has been reported that the mutation of *rpfE* had no detectable effects on the bacterial interactions with plants and no effects on the production of DSF [34,35]. In 2013, *rpfE* gene mutant strains were generated to elucidate the role of RpfE with respect to the *rpf* system in a Korean *Xoo* KACC10859m gene cluster [34]. The results suggested that the *rpfE* gene regulates the virulence of *Xoo* under different nutrient conditions without changes in DSF production.

Rpf-regulated processes have been identified using comparative proteomic findings, indicating changes in the abundance of several functional proteins in the *rpfC*, *rpfG*, and *rpfF* mutants. An analysis of the regulatory influence of individual Rpf proteins supported a previous conclusion based on transcriptomics [36].

### 2.2. DSF-Family Signals

Another representative class of signaling molecules is the QS system of Gram-negative bacteria, which has attracted considerable interest in DSF. In *Xcc*, the *rpf* gene clusters related to pathogenicity were initially identified to influence the production of EPS and extracellular enzymes [37]. Interestingly, when the *rpf* gene clusters were silenced, the production of extracellular enzymes decreased. However, protease and endoglucanase activities can be restored when an *rpfF* mutant is introduced in proximity to its wild-type parental strain. Therefore, it is speculated that small molecular signals, called DSFs, may induce the synthesis of extracellular proteases associated with *rpfF*. Moreover, the DSF activity was preliminarily detected using a protease assay, which involved the observation of a clear zone on a skimmed milk agar plate [37]. For further DSF activity detection, DSF biosensor strains have been established, which allow for a semi-quantitative determination [25]. DSF was isolated, purified, and identified from *Xcc* culture supernatants of *rpfC* mutants as *cis*-11-methyl-2-dodecenoic acid [38]. In addition, newly identified DSF-family signals were extracted from *Xoo* for further improvements in in vitro methods [39], which are as follows: *cis*-2-dodecenoic acid (BDSF), *cis*, and *cis*-11-methyldodeca-2,5-dienoic acid (CDSF). BDSF was originally identified in *Burkholderia cenocepacia* as being involved in the regulation of motility, biofilm formation, and virulence [40].

DSF-family signals are mostly present in *Xanthomonas* spp., which does not mean that DSF signals are restricted to *Xanthomonas*. On the contrary, it allows for essential insight into the broader significance of the DSF-family signaling systems in the bacterial world [41]. DSF signals or structurally related molecules are also produced by unrelated bacteria, such as *Burkholderia cenocepacia*, *Xyllela fastidiosa*, and *Stenotrophomonas maltophilia*, to coordinate expressions of virulence genes [40,42,43]. *Ralstonia solanacearum* not only produces AHL signals to regulate virulence, but it can also produce signal molecules close to the DSF family, suggesting that *Ralstonia solanacearum* has two ways to regulate virulence [44]. The human pathogen *Pseudomonas aeruginosa* can even produce the DSF-like molecule *cis*-2-decanoic acid to mediate inter- and intraspecies communications [45].

DSF exerts multifaceted effects on the invasion plants. In addition to the regulation of bacterial virulence via intra-communications [39], it has recently been reported that DSF can elicit plant immunity and participate in interspecies communication [46]. Plant actin is an important component of a plant’s cytoskeleton, and its assembly can be attenuated due to negative regulation of formin–formin interactions via DSF [47]. Additionally, DSF will be conducive to the competitive capability of *Xcc* against *Bacillus thuringiensis* through the modulation of *ftsZ* in relation to cell division [48]. These findings indicate the significant importance of DSF signaling in the bacterial world.

### 2.3. The QS Pathway in Xanthomonas

**Figure 2:**
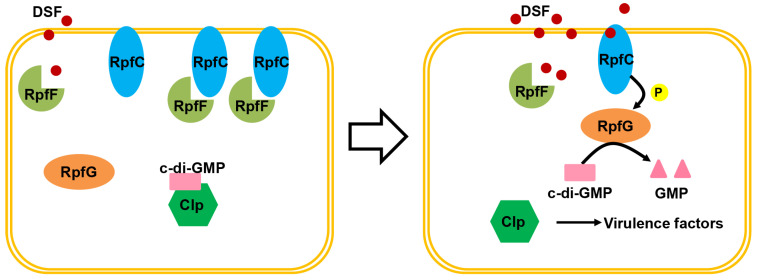
Schematic representation depicting the pathway of RpfF/DSF quorum sensing.

DSF-mediated QS has attracted much attention, and the scheme of the regulatory system by and large has been studied [21,49]. Generally, DSF is produced, detected, and transduced by Rpf proteins located upstream of the QS. Synthase RpfF is significant for the biosynthesis of DSF signals, and a hybrid sensor kinase RpfC negatively controls DSF synthesis due to protein–protein interactions with RpfF, which, conversely, can be responsible for DSF perception through H198 autophosphorylation [32]. As shown in Figure 2, at low cell densities, the catalytic activity of RpfF is hindered by its binding to RpfC, while only a few DSF signals are produced by the low levels of free RpfF. In addition, a second messenger, cyclic di-GMP, binds to the global transcription factor (Clp) and prevents the binding of Clp to the promoters of genes encoding virulence factors, including those encoding virulence factors downstream. At high cell densities, RpfC detaches from RpfF and combines with RpfG to form a two-component regulatory system, which is a mechanism of signal perception and transduction. The RpfC sensor can detect the DSF signal that is largely synthesized by the released RpfF, whereas the sensor mechanism is still unclear. Subsequently, the signal is transferred by RpfC to the response regulator RpfG using a phosphorelay mechanism, and RpfG activity as a cyclic di-GMP phosphodiesterase is activated. Finally, the free forms of Clp dominate, and, as a consequence, the decrease in cyclic di-GMP levels drive gene expression.

## 3. DSF Biosynthase

### 3.1. DSF Synthesis

RpfB, a fatty acyl-CoA ligase, was initially thought to be involved in DSF biosynthesis [40]. In the genome, *rpfB* and *rpfF* are adjacent, and their mutants can reduce DSF production. Therefore, it was speculated that both genes are important for DSF synthesis. As for the corresponding encoded enzymes, the RpfF enzyme catalyzes the DSF precursor produced by RpfB. Subsequently, RpfB activity is implicated in the turnover of DSF, whereby bacteria can exit from the QS phase [49,50]. At low cell densities, the complex of c-di-GMP and Clp suppresses *rpfB* expression. However, *rpfB* is expressed at high cell density concentrations, and RpfB switches to DSF signals. At the transcriptome level, DSF biosynthesis is overproduced in the presence of the *rpfC* deletion and is reduced in the presence of overexpression of the REC domain [44]. The REC domain blocks the putative substrate from binding to the Lock RpfF protein in an inactive state; thus, RpfC negatively controls DSF production [51].

**Figure 3:**
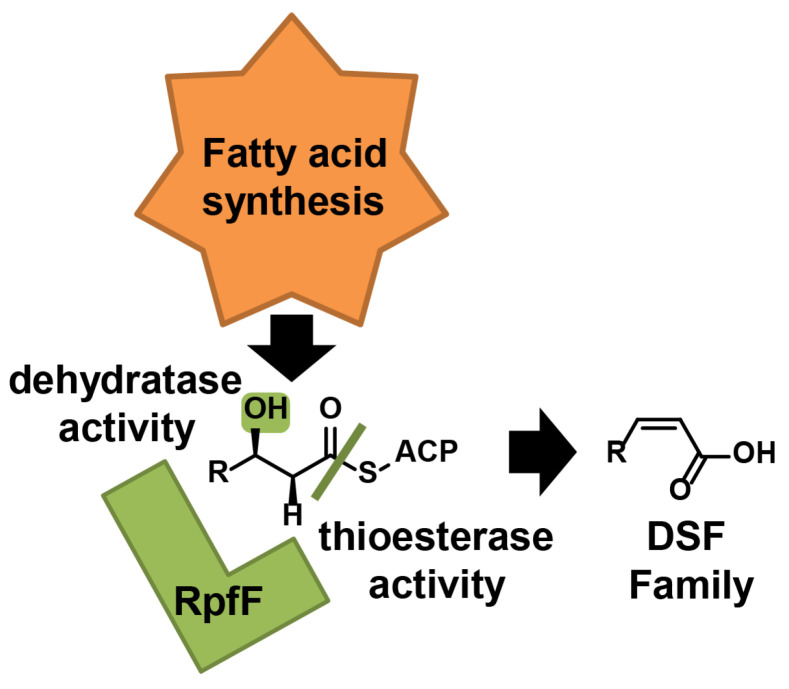
Schematic model for the process of DSF synthesis catalyzed by RpfF.

DSF biosynthesis is associated with FabH, and DSF signals can be modulated by the FabA–FabB pathway during the fatty acid synthesis process [52,53]. Substrates of 3-hydroxyacyl-ACP with different length chains are synthesized as DSF precursors via the fatty acid synthesis cycle and are then catalyzed by the RpfF enzyme to form DSF-family signals, such as DSF, BDSF, and IDSF [22,54]. In particular, two enzyme activities of RpfF have been identified [42]. As illustrated in Figure 3, firstly, RpfF exerts dehydratase activity, converting 3-hydroxyacyl-ACP into *cis*-2-acyl-ACP. Furthermore, RpfF may also cleave the ACP-thioester bond to release holo-ACP and generate DSF-family signals, further indicating the presence of thioesterase activity. Additionally, the RpfF enzyme in *Xyllela fastidiosa* has been shown to catalyze the synthesis of DSF through a similar process that involves the following two steps: catalysis of the formation of a double bond and then hydrolysis of the thioester bond [31].

Host plant metabolites, such as sucrose and glucose, may be efficiently utilized to boost DSF signal synthesis to improve the pathogenicity of *X. campestris* pv. *campestris* [55]. Meanwhile, after adding salicylic acid, the bacterial RpfB activity increases, leading to DSF signal turnover [56].

### 3.2. RpfF Protein

Understanding the structure and active sites of the RpfF protein will contribute to the development of novel inhibitors. To our knowledge, some studies have examined the crystal structure and performed homology modeling of the RpfF protein.

**Figure 4:**
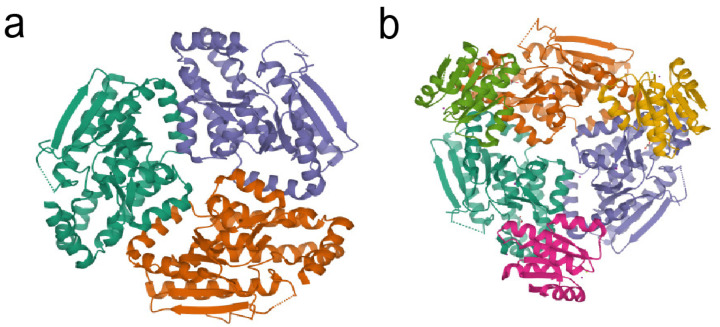
(**a**) 3D structure of the RpfF protein from *Xanthomonas campestris* pv. *campestris.* (PDB ID: 3M6N) (**b**) Crystal structure of RpfF, complexed with the REC domain of RpfC from *Xanthomonas oryzae* pv. *oryzae*. (PDB ID: 3M6M). The REC domain is shown in bottle-green, bright orange, and purplish red. The pictures are from the https://www.rcsb.org website accessed on 1 November 2022.

In 2010, Cheng et al. obtained the crystal structure of the RpfF protein through co-expression of both the RpfF and REC domains of RpfC in *E. coli* [51]. As illustrated in Figure 4, the RpfF with an N-terminal *α*/*β* spiral core domain and a C-terminal *α*-helical region belongs to the enoyl-CoA hydratase/isomerase family. By means of sequence alignment, it has also been determined that RpfF harbors two conserved glutamate residues, Glu141 and Glu161, corresponding to enoyl-CoA hydratase, but the residues corresponding to the isomerase are not required for catalysis. As a consequence, RpfF is likely a hydratase and not an isomerase. Meanwhile, the important role of the glutamate residues has been shown to involve DSF biosynthesis.

In 2012, the 3D structure of the RpfF protein from *Xoo* was predicted through a homology modeling method in silico using DSF synthase from *Xcc* as a template [57]. Consistent with previous studies by Cheng et al., the presence of a hydrophobic pocket in RpfF probably represents a docking site for a DSF precursor. Active site prediction studies showed that RpfF has 37 pockets, and, in particular, ID 37 could be a potential cavity, which contains approximately 20 amino acid residues. These significant residues are also highly conserved between RpfF and enoyl-CoA hydratases, including Glu141 and Glu161. The hydrophobic residues Met170 and Trp258 may come into contact with the DSF precursor molecule. Leu136, Gly137, Gly138, Gly85, and Leu276 may coordinate substrate binding and catalytic activity.

With regard to the research processes in non-*Xanthomonas*, RpfF proteins are structurally similar to members of the crotonase superfamily and have two highly conserved catalytic glutamate residues located at their active pockets [42]. A crystallization and a preliminary X-ray diffraction characterization of RpfF from *Stenotrophomonas maltophilia* have been reported [58].

On the basis of the above interesting results, RpfF is an important functional protein in *Xanthomonas* that plays a key role in DSF-mediated QS and has a highly conserved active site associated with DSF biosynthesis. Its structure is also conserved in various bacteria, and its functions are consistent. Overall, the structural information on the RpfF enzyme has increased its potential as an interesting target and raised the possibility of structure-based drug designs.

### 3.3. RpfF Inhibitors

**Figure 5:**
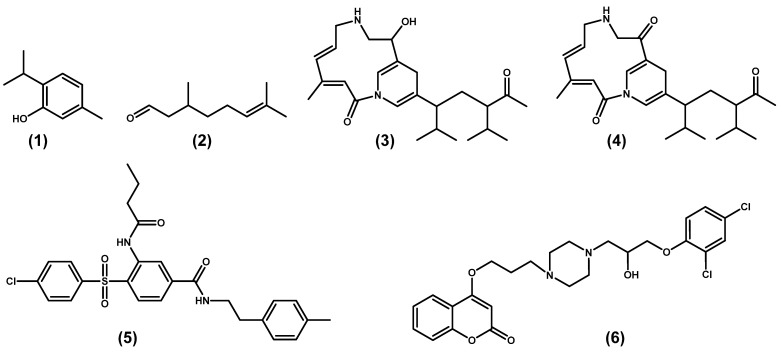
Chemical structure of reported RpfF inhibitors: (**1**) thymol, (**2**) citronellal, (**3**) chumacin-1, (**4**) chumacin-2, (**5**) rifampicin analogues, (**6**) and coumarin derivative.

Research on DSF-mediated QS is of significance for the control of plant diseases. RpfF may represent a potential antibacterial target, but little is known about inhibitors targeting RpfF. Among the current antibacterial studies, *Xoo* is a major research focus for the plant pathogen *Xanthomonas*. To date, research into its mechanism has revealed that its inhibitory actions entail a reduction in virulence factors, inhibition of QS-related genes, and suppression of *rpfF* genes and their encoded enzymes. The reported RpfF inhibitors were displayed in Figure 5 and listed in Table 1.

In 2017, thyme oil was reported to influence virulence factors, such as biofilm and extracellular enzymes, swimming and swarming with unaffected conditions of bacterial growth and metabolic activity under the control of *Xoo* [59]. Further authenticated by qRT-PCR, thyme oil exerted an inhibitory effect on gene expression, particularly on the *rpfF* gene. As HPTLC analysis results showed, a significant reduction in the synthesis of DSF and BDSF signaling molecules was observed after treatment with thyme oil. Thus, thymol (molecule 1) was identified as a major component of thyme oil for studies investigating molecular docking with the RpfF protein. Thymol was found to form potential hydrogen bonds with some residues of catalytic importance, such as Glu161 and Gly169, in the putative RpfF binding pocket.

In 2018, kaffir lime oil (KLO) was found to act as an anti-virulence agent in the context of normal bacterial growth for controlling *Xoo* [60]. Among the major components of KLO, citronella (molecule 2) showed excellent activity for inhibiting biofilm formation, swimming and swarming activities, and extracellular plant cell wall degrading enzymes. Gene expression analysis showed downregulation in transcript levels of genes associated with virulence factors and *rpfF*. Molecular docking analyses indicated an interaction between citronella and the RpfF protein, in which the molecule formed probable hydrogen bonds with important catalytic residues (GLY169 and TRP258).

In 2019, two bioactive compounds produced by the *Pseudomonas aeruginosa* strain CGK-KS-1 showed excellent antibacterial activities for *Xanthomonas* and were named Chumacin-1 (molecule 3) and Chumacin-2 (molecule 4), respectively [61]. Further mechanistic studies revealed that they inhibited the production of a QS signaling factor and suppressed xanthan gum secretion and biofilm formation by various *Xanthomonas* pathovars. Chumacin-1 and Chumacin-2 produced by the *Pseudomonas aeruginosa* strain CGK-KS-1 inhibited DSF ion activity in *Xoo*. The results showed that these extrolites could bind and inhibit RpfF proteins, and their interactions with RpfF exhibited good docking scores and binding energies.

In 2020, Srilatha et al. predicted the 3D structure of the RpfF protein using homology modeling and docked rifampicin analogues within the active site of RpfF by virtual screening in order to identify potent inhibitors [62]. Some of the screened compounds from the ZINC database had higher energies and lower toxicity than the reference compound rifampicin. Compound **5** was predicted as the optimal molecule. Moreover, active site residues of importance were identified, such as HIS-118, HIS-147, THR-148, ARG-179, ASP-207, ARG-240, and THR-244. In the same year, Mishra et al. discovered that silver nanoparticles exerted inhibitory effects on the *rpf* gene, but the actual biological activities of the compounds were not investigated further [63].

**Table 1:** Overview of reported RpfF inhibitors.

Inhibitor	Origin	Mechanistic Action	Reference
Thymol	Major component of Thyme oil	Formation of potential hydrogen bonds with some residues Glu161 and Gly169 in the putative RpfF binding pocket; downregulation of *rpfF* gene; reduction of DSF and BDSF.	Singh et al. [59]
Citronellal	Major component of Kaffir lime oil	Down regulation in transcript levels of *rpfF*; formed probable hydrogen bonds with important catalytic residues (GLY169, TRP258).	Singh et al. [60]
Chumacin-1	Pseudomonas aeruginosa strain CGK-KS-1	Inhibition of the production of DSF; suppressed the xanthan gum secretion and also inhibited the biofilms formed; interaction with the residues of RpfF pocket.	Kanugala et al. [61]
Chumacin-2			
Rifampicin analogues	Virtual screening form ZIN Database	Docked into the active site of the RpfF protein.	Srilatha et al. [62]
Coumarin derivative	Synthesis based on derivation of natural product	Disturbed biofilm formation; suppressed bacterial virulence factors and production of DSF; reduced expression of *rpfF* gene.	Feng et al. [64]

In 2022, a series of coumarin derivatives containing isopropanolamine units were designed as anti-QS agents and evaluated for their QS interference ability [64]. After a treatment with compound **6**, the results revealed a marked decrease in significant virulence factors, including biofilm formation, bacterial motility, levels of extracellular enzymes, and a notable reduction in DSF signal production and *rpfF* gene expression.

Based on the above-mentioned studies, the RpfF inhibitors can be divided into the following three categories: natural products, synthetic compounds, and silver inorganic materials. Among the natural products, compounds **1** and **2** were first verified from an oil extract, and their interaction with RpfF was demonstrated by molecular docking studies. Compounds **3** and **4** were isolated from non-*Xanthomonas* spp. To select new molecules to target the RpfF protein, engineering natural products based on a ramification strategy and high-throughput screening tactics were effective approaches. Compound **5** was selected from 2500 rifampicin analogues. In addition, some antibacterial agents could repress the expression of the *rpfF* gene or interfere with DSF signals, which indicated that this class of compounds may be potential RpfF inhibitors, such as silver nanoparticles and compound **6**.

## 4. Conclusions

DSF-mediated QS has been newly recognized as a major component of the pathogenicity network in *Xanthomonas*, and it can be considered a crucial target for suppressing plant bacterial diseases. During the drug design process, enzymes are a class of targets that need to be highly valued. Due to their high specificity, enzymes, which are involved in catalytic reactions associated with disease, can be an important potential target. Therefore, the ability to produce DSF makes RpfF a possible target against *Xanthomonas*. As for the design of antibacterial agents, the putative substrate-binding pocket and amino acid residues crucial for DSF synthesis also deserve closer attention. Although progress has been made in the exploration of DSF-mediated QS inhibitors in recent years, there are still many unknown active compounds targeting the signal synthetase RpfF that need to be discovered. Currently, the reported inhibitors have been identified as essential oils or derivatives of natural products, which are providing researchers with potential design ideas. More specifically, studying their vital mechanisms in antibacterial activity may make it possible to target the RpfF protein for the treatment of infections involving plant pathogens, especially with regard to pathogenicity and pesticide resistance.

In the past few years, QS inhibition strategies have made significant progress in the field of pesticides. As the crucial enzyme in the QS system, the signal synthetase RpfF can be regarded as a promising target against *Xanthomonas*. Although reported RpfF inhibitors are extremely limited, the design and discovery of RpfF inhibitors remain a challenge for the development of new agrochemicals. Therefore, future research should be directed to focus on addressing the following problems: (1) QS for crop protection is highly sought after by researchers, but the underlying molecular mechanisms of DSF-mediated QS are not well understood. (2) The DSF-binding pocket is a crucial target site; however, the challenge of designing effective RpfF inhibitors remains. (3) Although there are some RpfF inhibitors that may provide lead compounds for discovering new active molecules, an effective and diverse screening strategy is still lacking. Using strategies including natural product derivatization, computer-assisted drug design, and biomimetic synthesis may improve the operational efficiency of RpfF-targeted agrochemical development. Overall, the potential role of *rpfF* and RpfF inhibitor development in crop protection is worthy of continuous exploration by researchers.

## Data Availability

Not applicable.

## Abbreviations

SDH	succinate dehydrogenase
PK	pyruvate kinase
*Xcc*	*Xanthomonas campestris* pv. *campestris*
*Xoo*	*Xanthomonas oryzae* pv. *oryzae*
QS	quorum sensing
AIs	auto-inducers
AHL	*n*-acyl homoserine lactones
DSF	diffusible signal factor
Rpf	regulation of pathogenicity factor
FCL	fatty acyl-CoA ligase
HR	hypersensitive response
hrp	hypersensitive response pathogenicity
ACP	acyl carrier protein
KLO	kaffir lime oil
